# Secretomes from Conventional Insemination and Intra-Cytoplasmic Sperm Injection Derived Embryos Differentially Modulate Endometrial Cells *In Vitro*

**DOI:** 10.1007/s43032-024-01504-z

**Published:** 2024-03-12

**Authors:** Ameya Jijo, Itti Munshi, Shubhashree Uppangala, Rithika Rajendran, R. Vani Pratap LakshmiKumar, Guruprasad Kalthur, Borut Kovacic, Geetanjali Sachdeva, Satish Kumar Adiga

**Affiliations:** 1 Centre of Excellence in Clinical Embryology, Department of Reproductive Science, Kasturba Medical College, Manipal Manipal Academy of Higher Education, Manipal, 576 104 India; 2 Cell Physiology and Pathology Laboratory, Indian Council of Medical Research - National Institute for Research in Reproductive and Child Health, Mumbai, India; 3 Division of Reproductive Genetics, Department of Reproductive Science, Kasturba Medical College, Manipal. Manipal Academy of Higher Education, 576 104, Manipal, India; 4 Department of Data Science, Prasanna School of Public Health, Manipal Academy of Higher Education, Manipal, 576104 India; 5 Department of Reproductive Medicine and Surgery, Kasturba Medical College, Manipal. Manipal Academy of Higher Education, 576 104, Manipal, India; 6 Division of Reproductive Biology, Department of Reproductive Science, Kasturba Medical College, Manipal. Manipal Academy of Higher Education, 576 104, Manipal, India; 7 Department of Reproductive Medicine, University Medical Centre, Maribor, Maribor Slovenia

**Keywords:** Embryo, endometrium, Fertility, ICSI, Implantation, Insemination, Integrin, IVF, Secretome

## Abstract

Conventional Insemination (CI) and Intra-Cytoplasmic Sperm Injection (ICSI) are routinely used insemination methods in clinical Assisted Reproductive Technologies (ART) settings. However, the existing data on the developmental competence and implantation potential of CI and ICSI derived embryos are not unequivocal. This prospective study on 23 patients undergoing ART treatment explored whether the secretomes of CI- and ICSI-derived embryo differentially alter the expression of integrins (α_v_ and β_3_ integrin) and MUCIN-1 (MUC-1) in a human endometrial epithelial cell line (Ishikawa). Immunocytochemical data demonstrated that the secretome of CI-derived top quality (GI) embryos induced higher (p < 0.05) expression of ɑ_v_ β_3_ compared to sibling ICSI derived G1 embryos in Ishikawa cells. Though, relative levels of the transcript for MUC-1, anti-adhesion molecule did not show a significant difference between the study groups, immunocytochemical analysis demonstrated significantly (p < 0.0001) higher expression of MUC-1 in cells treated with ICSI-derived embryo secretome, compared to that treated with CI -derived embryo secretome. These results suggest that secretomes from CI and ICSI embryos differentially modulate the endometrial cells *in vitro*. This hints at differences in the ability of CI- and ICSI- derived embryos to alter endometrial profile.

## Introduction

Conventional Insemination (CI) and Intra-Cytoplasmic Sperm Injection (ICSI) are routinely used insemination methods in clinical Assisted Reproductive Technologies (ART) settings. Several reports recommend ICSI for attaining higher fertilization rate and better embryo yield to improve the cumulative pregnancy rate [1–5]. Nonetheless there exist sufficient data to suggest that ICSI is not helpful in achieving higher pregnancy or live birth rates, compared to CI [6–10]. Yet, ICSI is preferred insemination technique, in about 70% of fresh ART cycles, indicating the overuse of this method [8, 11–13].

Reports on the developmental competence and implantation potential of CI- and ICSI- derived embryos are contradictory [8, 14–19]. ICSI is reported to have a better clinical outcome than CI in couples with non-male factor infertility [20–23]. Several meta-analyses have inferred that implantation and live birth rates from CI-derived embryos are higher, compared to that resulting from ICSI [6, 17, 19], A recent study has also shown that compared to CI, ICSI results in a lower fertilization rate and lower rate of euploid embryos [24].

Bi-directional communication between blastocyst and endometrium is an important requisite for successful implantation and pregnancy. Studies using rodent [25–27] and primate models have demonstrated the ability of embryonic signals to modulate the endometrium [28–30]. The secretome of human embryos is also reported to modulate the endometrial cells at molecular level [31–33]. Further, human embryo secretome was found to promote proliferation of endometrial cells and modulate the expression of implantation- related genes [34]. However, these studies employed pooled spent culture media from embryos, irrespective of their quality. Hence it was not clear whether the alterations observed in the endometrial epithelial cells were induced by only good quality embryos or whether these changes were observed due to certain embryonic products, that are secreted independent of their quality.

A few strides have been made to analyse Spent Culture Medium (SCM) of CI- and ICSI- derived embryos to explore whether these embryos differ at molecular level. SCMs of CI- and ICSI- derived embryos from patients with moderate male factor infertility did not differ in terms of their metabolic profiles [35]. Our recent NMR-based study, also found metabolomic signatures of CI and ICSI derived embryos from non-male factor patients undistinguishable [36]. However, it is likely that NMR spectroscopy, probably failed to identify the low-abundant metabolites, differentially abundant in the secretome of CI- and ICSI- derived embryos [37, 38]. Though, developmentally and morphologically CI- and ICSI- derived embryos are comparable [39], differences have been reported in their transcriptomes, proteomes [24, 40], and epigenomes [41]. These alterations may have implications on the implantation potential of CI- and ICSI- derived embryos. It is likely that the secretomes from CI and ICSI derived embryos differentially trigger various signalling pathways and thereby differentially modulate the expression of implantation-related genes in endometrial cells.

To the best of our knowledge, there is no study that has employed a cell-based assay to compare the competency of CI- and ICSI- derived embryos to modulate endometrial epithelial cells. Hence we made an attempt to explore whether CI and ICSI- derived embryo secretomes differentially alter the expression of integrin ɑ_v_β_3_ and MUCIN-1 in human endometrial epithelial cell lines. Integrin ɑ_v_β_3_ is a receptivity marker that shows increased expression in the mid-secretory phase of menstrual cycle. Integrin is known to play a role if embryo attachment, angiogenesis as well as placental invasion into the maternal vasculature [42]. MUC-1, also receptivity marker, regulates implantation by inhibiting cell-to-cell adhesion. Local reduction of MUC-1 expression at attachment sites allows the blastocyst to implant and, MUC-1 expression is reported to be critical for the selection of high-quality embryos [43]. It was envisaged a comparison of the ability of secreted factors from CI- and ICSI- derived embryos to modify the endometrial epithelial cells will indirectly establish whether the molecular profiles of that CI- and ICSI- derived embryos are similar or dissimilar.

## Methods

### Study subjects

A total of 23 couples undergoing ART treatment at the University's infertility clinic were included in this prospective study. Approval from Institutional Ethics Committee (IEC1: 297/2022) was taken prior to the initiation of the study. Patients who signed the informed consent and qualified the following criteria were included in the study: i) women < 35 years of age ii) having no pelvic pathologies such as endometriosis or tubal abnormalities, as indicated by their medical history; ii) having regular menstrual cycles as disclosed in their medical history; iii) having no metabolic/endocrine system-related conditions, such as hyperprolactinemia or hypo/hyperthyroidism iv) partner’s semen characteristics classified as ‘normal’ as per the WHO 2010 reference range [44]. The demographic characteristics of the patients and clinical investigations details are summarized in Table 1.

**Table 1:** Patient’s demographics and clinical characteristics

Age-female (year ± SD)	31.13 ± 3.12
Age-male (year ± SD)	37.08 ± 2.98
Duration of infertility (year ± SD)	5.75 ± 1.86
Basal FSH (mIU/mL ± SD)	5.38 ± 1.89
Basal LH (mIU/mL ± SD)	5.81 ± 1.88
Basal estradiol (pg/mL ± SD)	44.84 ± 12.75
AMH (ng/mL ± SD)	6.55 ± 5.29
AFC (n ± SD)	17.47 ± 6.85
Duration of COS (days ± SD)	9.95 ± 1.02
Estradiol on day of trigger (pg/mL ± SD)	5580 ± 2660.49
LH on the day of trigger (mIU/mL ± SD)	2.57 ± 1.77
Progesterone on the day of trigger (ng/mL ± SD)	0.98 ± 0.66
Total sperm number (m ± SD)	136.93 ± 75.14
Total sperm motility (% ± SD)	60.39 ± 11.11
Sperm morphology (% normal forms ± SD)	12.09 ± 5.38
Sperm DNA damage (% ± SD)	9.5 ± 2.90

### Ovarian stimulation and oocyte retrieval

An antagonist protocol was performed during Controlled Ovarian Stimulation (COS). Recombinant FSH (rFSH, Gonal F®, Merck) was administered on day 2 of the cycle. Based on age, antral follicle count, and Anti-Mullerian Hormone level, initial dose of rFSH ranged from 225 to 450 IU/day. Thereafter, the rFSH dose was increased/decreased based on the ovarian response until the day prior to hCG administration. Pituitary down-regulation was accomplished by daily injection of the GnRH antagonist Citrotide ® 0.25 mg, (Merck), starting from Day 5 of the stimulation. Recombinant human chorionic gonadotropin (Ovitrelle® 250 mg, Merck Biopharma) was administered when at least four follicles had a mean diameter of 18 mm. Follicular aspiration was done under anesthesia using transvaginal ultrasonography. Aspirated oocyte cumulus complexes (OCC) were washed in Onestep medium (Vitromed GmbH, Germany; Cat No. V-OSM-20) and incubated at 37 °C under 6% CO_2_ for 2–3 h.

### Fertilization and embryo assessment

Split insemination was performed using CI and ICSI techniques by randomly assigning the sibling Oocyte Cumulus Complexes (OCC). Insemination droplet (80 µL) having 15,000 to 20,000 spermatozoa from the processed fraction of the ejaculate was overlaid with oil (Vitromed GmbH, Germany; Cat No V-OIL-P100). Conventional Insemination (CI) was performed by transferring OCC to individual insemination droplet followed by co-incubation at 37 °C, 6% CO_2_, and 5% O_2_ in the MIRI® Multiroom incubator (ESCO Medical, Singapore). After 16–18 h, zona-bound spermatozoa and cumulus cells were completely removed by mechanical pipetting and multiple washing steps.

ICSI was performed by selecting single, motile, morphologically normal spermatozoon and injecting into a metaphase II oocyte using Olympus-Narishige workstation. Post ICSI, oocytes were washed and cultured individually in 30 µL Onestep medium droplet covered with oil. Fertilization evaluation was performed simultaneously 16–18 h after CI or ICSI and fertilized oocytes from each group were transferred to a freshly prepared 30 µL droplet of Onestep medium and incubated at 37 °C, 6% CO_2_, and 5% O_2_ in a MIRI® Multiroom incubator. On day 3, embryos were microscopically assessed from both the groups for cell number, blastomere regularity, and morphological defects as per the ESHRE consensus [45] and graded accordingly. Grade I embryos had stage-specific cell number and size, with < 10% fragmentation and had no multinucleation. Embryos were either selected for transfer or cryopreserved, based on the clinical indication.

### Spent embryo culture medium (SCM) collection

SCMs of those that developed into optimal embryos were collected carefully by aspirating 30 µL from the droplet without oil contamination and placed individually into labelled sterile cryovials, snap frozen in liquid nitrogen, and then stored at -80 °C [36]. One medium droplet without the embryo, maintained under identical conditions, was used as the medium control (MC) for every experimental group (CI or ICSI) from every patient.

### Maintenance of cell lines

Ishikawa endometrial epithelial cell line (Sigma-Aldrich) derived from human endometrial adenocarcinoma was used. Ishikawa cell line was propagated in complete Dulbecco’s Minimal Essential Medium (DMEM)/F-12 medium supplemented with 10% fetal bovine serum (FBS) (Thermo Fisher Scientific, USA; Cat No. 10082147), 100 units/mL penicillin and 100 µg/mL streptomycin (Gibco™, Grand Island, USA; Cat No. 15140122).

### Cell culture

Experiments related to cell culture, SCM treatment and subsequent analysis were performed at Indian Council of Medical Research (ICMR)-National Institute for Research in Reproductive and Child Health (NIRRCH), Mumbai. Ishikawa cells were seeded at a density of 1 × 10^4^ per well, in a 48- well culture plate (Cat No.150687) over a coverslip [46]. The cell density was kept uniform for all the experiments. Post 16 h, culture media was replaced with 240 µl of medium supplemented with 10 μl of SCM or MC. Cells were grown for 72 h and monitored regularly using an inverted phase-contrast microscope (Nikon eclipse Ti-S).

### Immunocytochemistry

Expression levels of a heterodimer of two integrins (ɑ_v_ and β_3_) and anti-adhesion protein, Mucin-1 (MUCIN-1) were assessed in Ishikawa cells treated with CI- or ICSI- derived SCMs. Cells grown on coverslips after 72 h treatment were fixed in 2% Paraformaldehyde (PFA), 0.05% glutaraldehyde and 120 mM sucrose for 30 min at room temperature and washed with 1 × PBS followed by blocking with 1% BSA for 30 min [34]. Cells were incubated with mouse antibodies against ɑ_v_ β_3_ integrin heterodimer (1:25), conjugated to Alexa Fluor® 488 (Santa Cruz Biotechnology Inc, USA; Cat No. SC-7312). In another set of experiments, cells were stained with mouse antibodies against human Mucin-1 (1:75) (Santa Cruz Biotechnology Inc, USA; Cat No. SC- 6827) for 16 h at 4 °C. After three 1 × PBS washes, cells stained with antibodies against MUC-1 were incubated with goat anti-mouse IgG conjugated to Alexa Fluor 488 (1:100) (Thermo Fisher Scientific, USA; Cat. No. A11001) for 1 h at 37° C. Finally, cells were stained with DAPI (4’, 6-Diamidino-2-phenylindole) (Roche, Basel, Switzerland; Cat No. 10236276001) and mounted with Vectashield antifade mounting medium (VECTASHIELD® Antifade Mounting Medium, USA; Cat. No. H-1000–10).

Images of immunostained cells were captured using confocal laser scanning microscope (Olympus, Germany) in XY plane using 60 × objective. Five fields for each sample were selected. Image analysis was performed by determining the intensities for immunoreactive antigens in Ishikawa cells using the image analysis software Image J. The raw integrated density (RawIntDen) per field was determined by calculating the average of the staining intensities of each cell in that particular field. Integrated optical density (IOD) for each sample was calculated as the mean of the RawIntDen of all the fields. Background settings were adjusted by observing the negative control wherein cells were incubated with 1 × PBS, instead primary antibody.

### Total RNA isolation, cDNA synthesis, and gene expression analysis

Total RNA was extracted from cells treated with SCMs from CI, ICSI or MC group using RNAqueous micro kit (Ambion, Life Technologies, USA; Cat No. AM1931) according to the manufacturer’s instructions. 1 µg of total RNA was reverse transcribed using random hexamers by high-capacity cDNA RT kit (Applied biosystems, USA; Cat No. 4368814) according to the manufacturer`s protocol. Quantitative real-time polymerase chain reaction (RT qPCR) was carried out using Premix Ex Taq kit (TaKaRa Bio, Japan; Cat No. RR390A), in StepOne™ Real-Time PCR System (Thermo Fisher Scientific, USA). 1 µL cDNA was used to determine the relative levels of *Integrin ɑv, Integrin β3* and *MUCIN-1* transcripts in cells treated with the SCMs from embryos, compared to MC treated embryos using Premix Ex Taq kit (TaKaRa Bio, Japan; Cat No. RR390A) QuantStudio™ 5 Real-Time PCR System (Thermo Fisher Scientific, USA). TaqMan assays (Thermo Fisher Scientific, USA) ITGB3 (Hd01001469_m1), ITGAV (Hs00233808_m1) and MUC1 (Hs0059357_m1) taqman assays were used. Transcript levels of integrin ɑ_v,_ integrin β_3_ and MUC-1 gene were normalized against the endogenous housekeeping gene 18S rRNA [47]. ΔC_t_ values were calculated as the difference in the threshold cycles between the target genes and reference genes ΔC_t_ = C_t_ (target gene) – C_t_ (reference gene) for each sample.

### Statistical analysis

The demographic and clinical characteristics of the patients enrolled are represented as mean ± standard deviation of the mean (mean ± SD) or median (IQR) after verification of the normality assumption (using the Shapiro–Wilk test). Univariate analysis of the embryological characteristics was implemented using GraphPad Prism 8 (GraphPad Prism software, CA, USA). Based on assessment of the normality assumption and the homogeneity of variance assumption (using the Levene's test) for the parameters across the groups, appropriate statistical inference methods were implemented using Jamovi 2.3.24 (graphical user interface for R programming). When both the assumptions are satisfied, Fisher's ANOVA with Tukey's post-hoc comparison was utilized to facilitate comparisons of parameters across multiple groups. Kruskal Wallis ANOVA with Dwass-Steel-Critchlow-Fligner post-hoc comparison was used in scenarios wherein a violation of normality and homogeneity of variance assumption was observed. The level of significance was set at 5% throughout the study. Furthermore, histograms were plotted using GraphPad Prism 8 (GraphPad Prism software, CA, USA). The assumption of data normality for correlation analysis was tested using Shapiro–Wilk test. Correlation analysis was performed using the spearman rank correlation coefficient since data was not distributed normally. The results were considered as statistically significant at *p* < 0.05. In the present study Fisher's ANOVA with Tukey's post-hoc comparison was used in the present study for the following parameters: for comparing transcript levels of integrin ɑ_v_, integrin β_3_ and MUC-1 between two groups. For Immunocytochemistry results, Kruskal Wallis ANOVA with Dwass-Steel-Critchlow-Fligner post-hoc comparison was used.

## Results

### Patient characteristics, fertilization outcome and embryo quality

Demographics and clinical characteristics of patients (n = 23) included in this study are shown in Table 1. Embryological data are presented in Table 2. Though the average number of oocytes used for insemination in the two groups was comparable, the fertilization rate was significantly higher in ICSI group (p < 0.05). The cleavage rate and embryo quality on day 3 were comparable between the two groups.

**Table 2:** Embryological characteristics

	CI	ICSI
Oocytes inseminated (mean ± SD)	9.56 ± 3.31	10.17 ± 1.64
Fertilization rate (% ± SD)	63.73 ± 21.69	79.26 ± 17.38^*^
Cleavage rate (% ± SD)	89.15 ± 16.57	91.95 ± 22.40
Day 3- good quality (Grade I) embryos (% ± SD)	40.07 ± 27.60	51.43 ± 26.15
Day 3- average quality (Grade II) embryos (% ± SD)	34.86 ± 24.80	26.40 ± 19.06
Day 3- poor quality (Grade III) embryos^**^ (% ± SD)	14.21 ± 15.68	14.11 ± 12.25

^*^p < 0.05 with the corresponding group

^**^ excludes embryos arrested at one cell stage

### SCM of human embryos upregulated the expression of integrin (ɑ_v_ β_3_) in Ishikawa cells

**Fig. 1:**
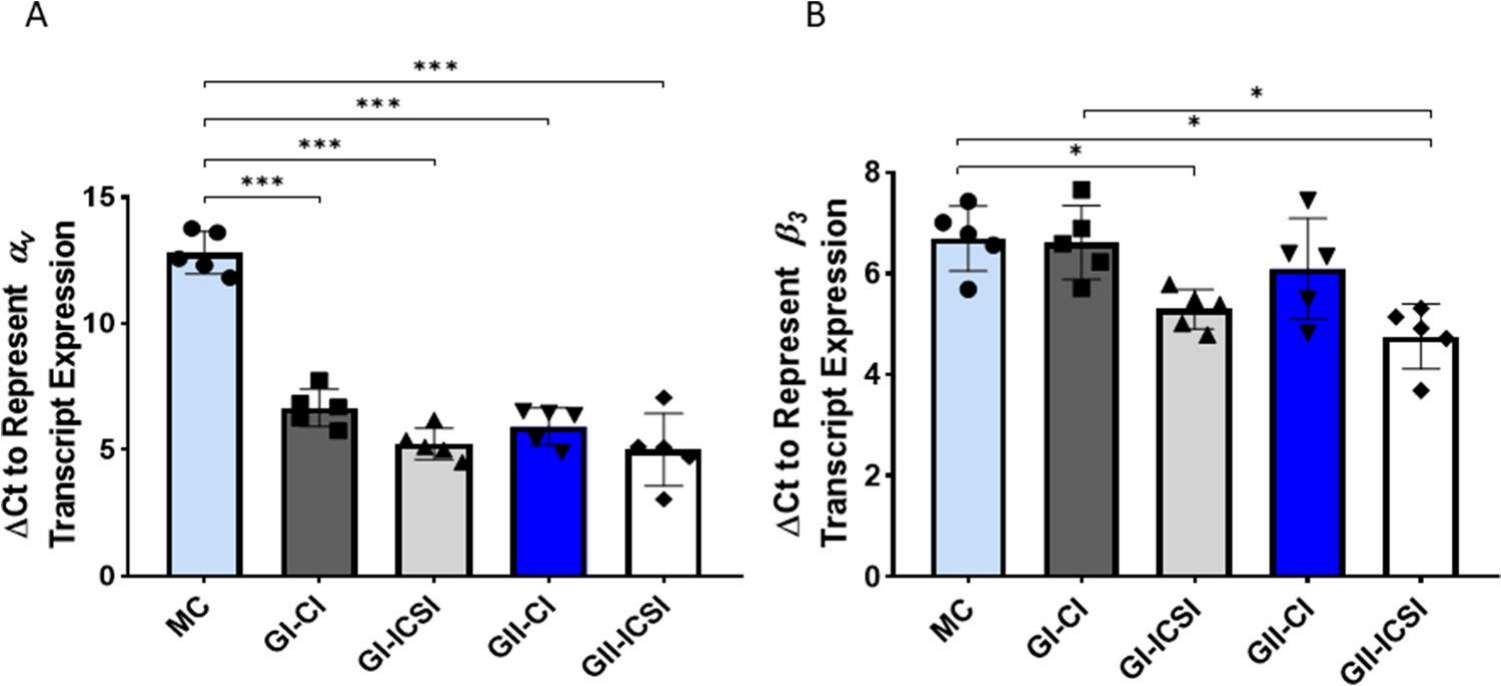
Relative levels of ɑ_v_ (**A**) and β_3_ (**B**) trancripts in Ishikawa cells treated with the secretomes (SCM) of human embryos derived (by CI and ICSI) of grade I and grade II (n = 5 each). Total RNA extracted from Ishikawa cells treated with media alone (MC) or SCM of CI/ICSI sibling embryos was converted to cDNA and amplified for assessing the levels of ɑ_v_ and β_3_ transcripts. 18S rRNA was used as a housekeeping gene. Taqman primer probes for the gene of interest (**ɑ**_**v**_** and β**_**3**_) and housekeeping gene 18S rRNA were used in real time RT-PCR assays. ΔC_t_ (threshold cycle) were determined. ΔC_t_ was calculated as C_t_ (gene of interest)- C_t_ (housekeeping gene). *p < 0.05; ***p < 0.0001

Relative levels of Integrin ɑ_v_ and β_3_ transcripts were assessed using Real-time qRT PCR of RNA extracted from Ishikawa cells treated with SCMs derived from CI or ICSI derived embryos of grade I or grade II (n = 5 each). Cells treated with embryo SCMs had significantly lower ΔC_t_ value for integrin ɑ_v_ compared to cells treated with media alone, indicating that the embryo secretome from both groups (ICSI- or CI- derived embryo) led to a significant by (p < 0.0001) higher expression of α_v_ transcript compared to the cells treated with media alone (MC). Further, this effect was observed irrespective of the type (CI/ICSI) or grade of embryo (Fig. 1A). Thus, a significant modulation in the levels of integrin ɑ_v_ transcript by embryo SCMs occurred irrespective of the embryo quality and insemination techniques. On the other hand, integrin β_3_ transcript levels did not differ significantly between MC and CI groups. Interestingly ICSI group showed significantly (p < 0.05) higher level of β_3_ transcripts compared to MC group.

**Fig. 2:**
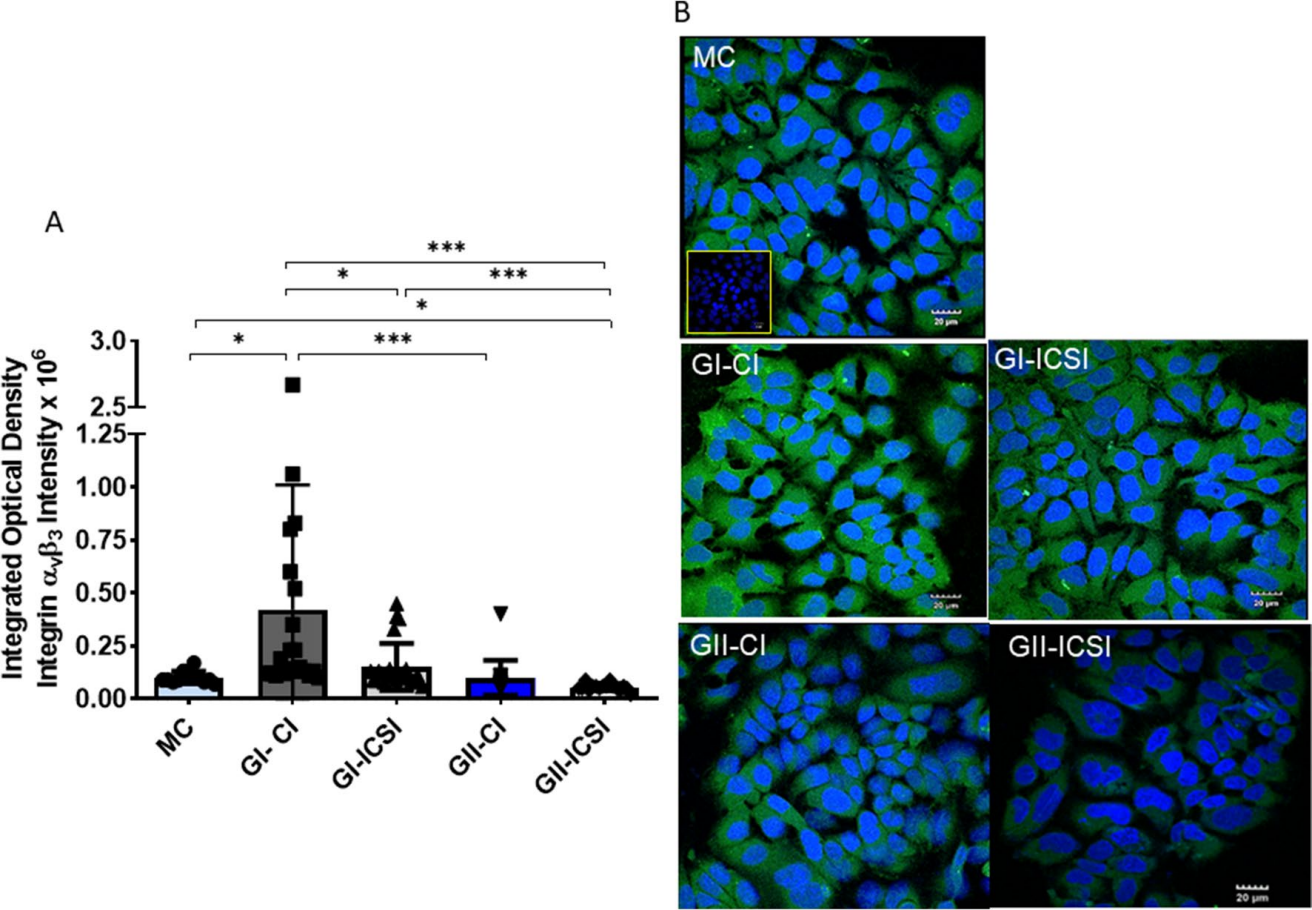
Immunolocalization of integrin (ɑ_v_β_3_) heterodimer in Ishikawa cells treated with SCM. **A** Relative cytoplasmic expression of integrin (ɑ_v_β_3_) in Ishikawa cells treated with SCM from grade I (21 GI-CI & 24 GI-ICSI) and II (16 GII-CI & 16 GII-ICSI) embryos derived from CI or ICSI techniques along with MC (n = 11). *p < 0.05; ***p < 0.0001. **B** Immunocytochemical localization to depict the localization of integrin (ɑ_v_ β_3_) heterodimer in Ishikawa cells treated with SCM of MC or GI and GII embryos derived from CI and ICSI

To validate these observations, immunolocalization of integrin (ɑ_v_β_3_) protein was carried out (Fig. 2). The cytoplasmic expression of integrin (ɑ_v_β_3_) in epithelial cells treated with the SCM from CI derived embryos (G1) was significantly (p < 0.05) higher than that detected in the MC treated cells (n = 11) (p < 0.05) (Fig. 2A). In contrast, integrin (ɑ_v_β_3_) expression was significantly (p < 0.05) lower in the ICSI group (II) as compared to MC group.

Further, when compared between the cultures stimulated with SCM from GI (21 GI-CI & 24 GI-ICSI) and GII (16 GII-CI & 16 GII-ICSI) embryos, GI embryo secretome group showed significantly (p < 0.0001) higher expression than that observed in GII group. Importantly, ICSI-derived GI embryo secretome resulted in a significantly (p < 0.05) lower expression of α_v_β_3_ heterodimer in Ishikawa cells compared to CI-derived GI embryos. Representative images of Ishikawa cells demonstrating expression of heterodimer integrin (ɑ_v_β_3_) are presented in Fig. 2B. Overall, the secretome of CI-derived embryo was found to induce higher expression of ɑ_v_β_3_ heterodimer compared to secretome of sibling ICSI-derived embryo.

**Fig. 3:**
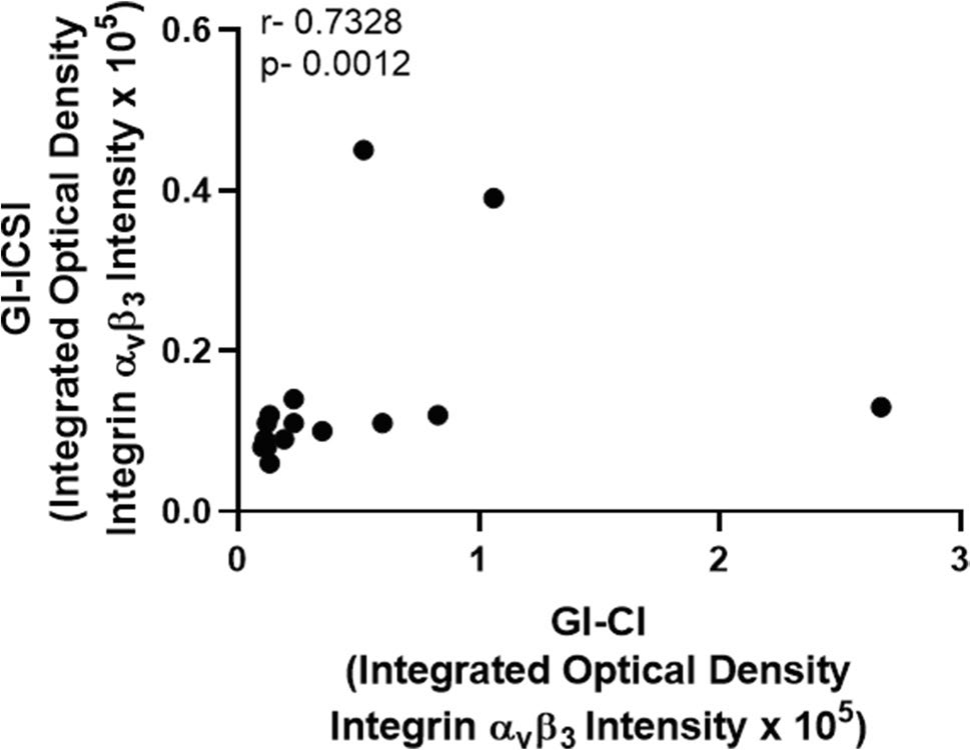
Correlation of Integrin (ɑ_v_β_3_) heterodimer expression observed in Ishikawa cells treated with the SCM of grade I sibling human embryos derived by CI and ICSI (n = 17 sibling embryo pairs). Integrated optical density of immunoreactive integrin (ɑ_v_β_3_) as indicator of relative cytoplasmic expression of ɑ_v_β_3_ in Ishikawa cell treated with SCM of grade I sibling human embryos derived by CI and ICSI technique. T Spearman Correlation Coefficient, r between the two groups was 0.7328. p < 0.001

Further, a significant (p < 0.001) positive correlation (Spearman correlation coefficient, r = 0.7328) was found for integrated optical density units for immunoreactive ɑ_v_β_3_ localization in Ishikawa cells between the SCMs from CI- and ICSI-derived sibling embryos (Fig. 3). Thus, treatment with the secretomes of CI- and ICSI-derived embryos from sibling oocytes led to higher expression of ɑ_v_β_3_ in Ishikawa cells. However, intensity of immunolocalized integrin (ɑ_v_β_3_) in endometrial cells treated with the SCM from CI-derived embryo was higher than that induced by the respective sibling ICSI-derived embryo.

### Human SCM induced expression of MUC-1 in Ishikawa cells

**Fig. 4:**
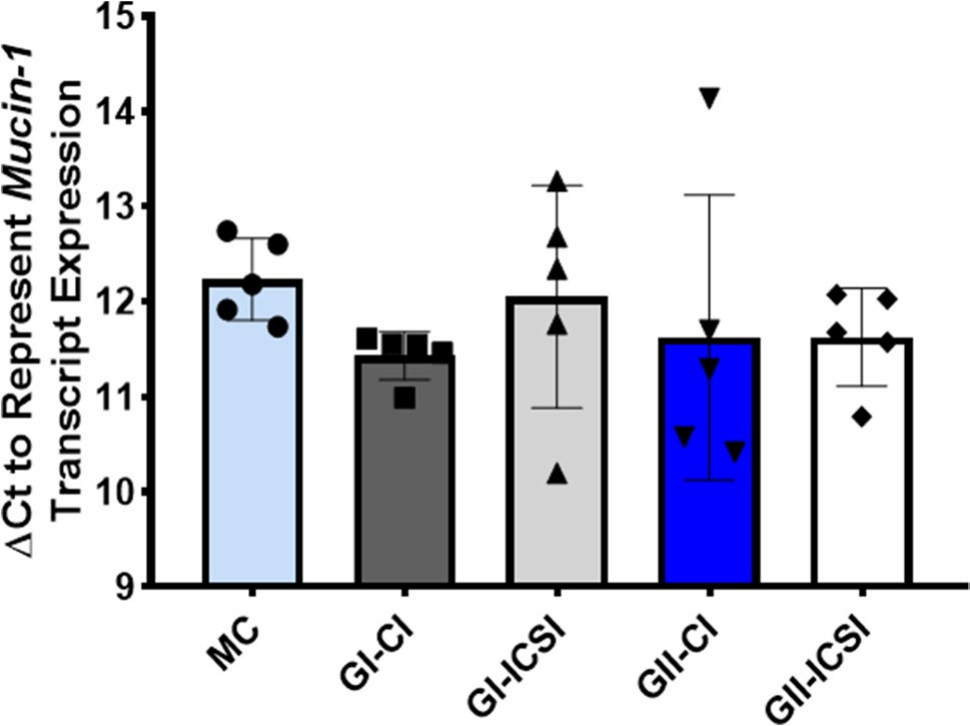
Relative levels of Mucin-1 (MUC-1) transcripts in Ishikawa cells treated with the SCMs of human embryos derived (by CI and ICSI) of grade I and grade II (n = 5 each). Total RNA extracted from Ishikawa cells treated with media alone (MC) or SCM of CI/ICSI sibling embryos was converted to cDNA and amplified for assessing the level MUC-1 transcript. 18S rRNA was used as a housekeeping gene. Taqman primer probes for MUC-1 gene and housekeeping gene 18S rRNA were used in real time RT-PCR assays. Δ C_t_ (threshold cycle) were determined. Δ C_t_ was calculated as C_t_ (gene of interest)- C_t_ (housekeeping gene)

**Fig. 5:**
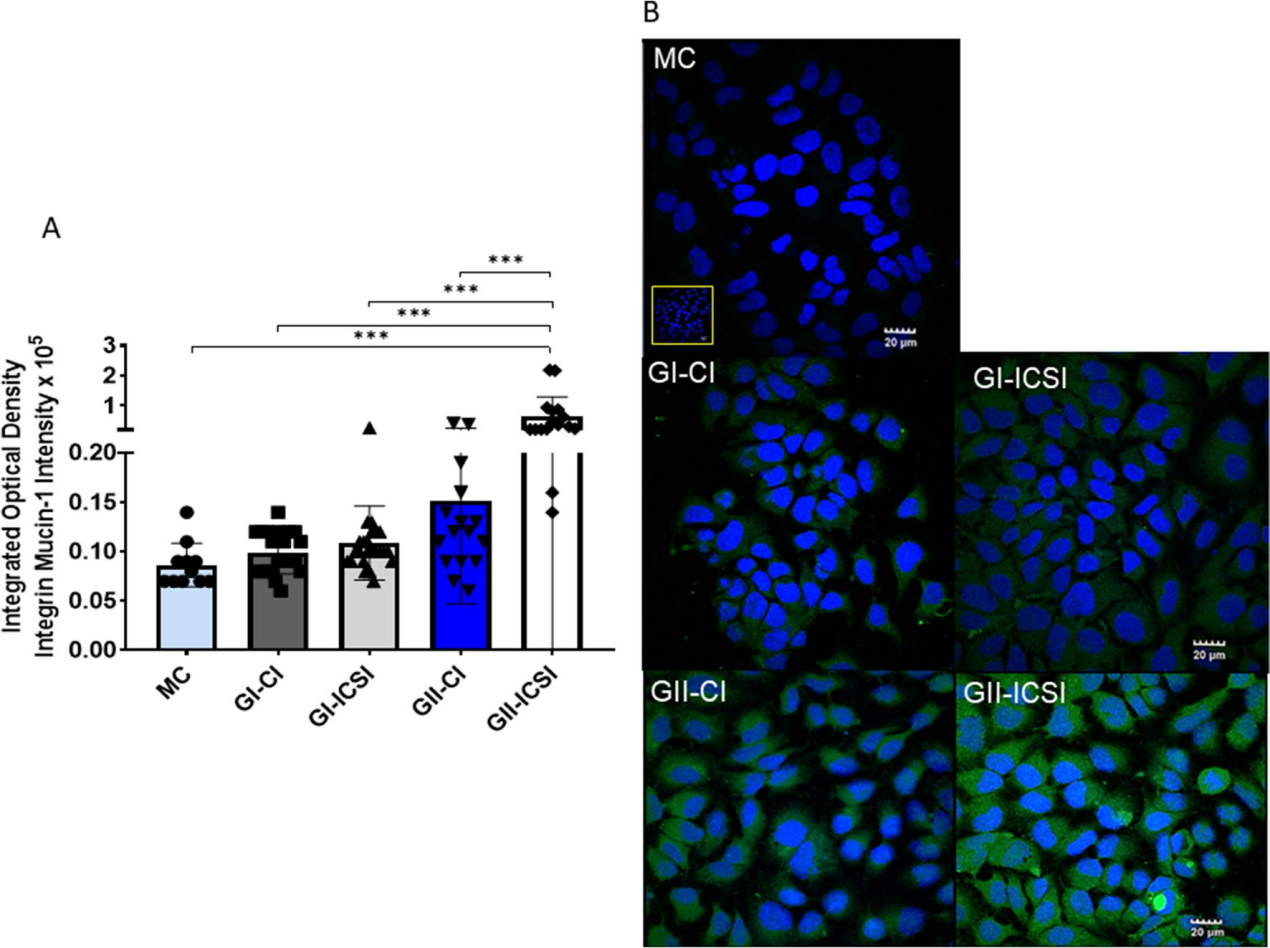
Immunolocalization of Mucin-1 (MUC-1) in Ishikawa cells treated with SCM. **A** Relative cytoplasmic expression of MUCIN-1 (MUC-1) in Ishikawa cells treated with SCM from grade I (21 GI-CI & 24 GI-ICSI) and II (16 GII-CI & 16 GII-ICSI) embryos derived from CI or ICSI techniques along with MC (n = 11), ***p < 0.0001. **B** Immunocytochemical localization to depict the localization of Mucin-1 (MUC-1) in Ishikawa cells treated with SCM of MC or GI and GII embryos derived from CI and ICSI

Relative expression of MUC-1 transcripts did not differ significantly between the study groups of grade I and grade II embryos secretome treated cells (n = 5 each)(Fig. 4). However, immunocytochemistry data showed four-fold higher expression of MUC-1 in Ishikawa cells treated with SCM from GII-ICSI group compared to corresponding MC, GI-ICSI(21 GI-CI & 24 GI-ICSI) and GII-CI (16 GII-CI & 16 GII-ICSI) groups (p < 0.0001; Fig. 5A). Representative images of cells immunostained for MUC-1 are shown in Fig. 5B.

## Discussion

Currently there is no consensus on the quality, implantation rates and pregnancy outcome of cycles wherein embryos derived by different insemination techniques (CI and ICSI) are transferred [20–23, 48]. Nonetheless, it is well established that autocrine and/or paracrine factors released by an embryo influence the surrounding microenvironment [49, 50] and also endometrial profile. We hypothesise that the secretomes of CI and ICSI derived embryos may have differential ability to modulate endometrial epithelial cells. Towards this sibling embryo secretomes were used to minimize confounding factors. Our data suggested differential response of endometrial epithelial cells to the secretomes of CI- and ICSI- derived embryos.

Results from the present study demonstrated that fertilization rate was higher in the ICSI group. This is in contrast to the other reports where in higher fertilization rates were observed when CI was used in non-male factor infertility patients [2, 51, 52]. It is important to note that sibling oocytes were used to ensure that all *in-vitro* conditions except insemination techniques were kept identical between the two groups in this study.

*In vitro* studies have shown that human SCM secretome influences the endometrial cells [31–33] either by increasing the cell proliferation or by modulating the expression of implantation-related genes [34, 53]. Clinically, embryo transfer along with its SCM has been shown to significantly improved the pregnancy outcome [53]. Hence, it is possible that the embryo secreted factors may favour embryo-endometrial interactions and improve implantation. Though the exact mechanism is not completely elucidated, Giacomini et al., (2017) demonstrated human embryos secrete extracellular vesicles that can be taken-up easily by the endometrial cells.

Integrins cell adhesion molecules, are known to have well-established role in cell invasion, migration, and embryo- endometrial interactions [54–56]. Integrins play a key role in mammalian implantation [57–61]. Therefore, we opted to investigate whether endometrial integrin expression is differentially altered in response to the secretomes of CI and ICSI derived embryos. Ishikawa cell line was selected as it has characteristics of both glandular and luminal epithelium [62] and is widely considered a good model for studying normal endometrial function. Importantly, Ishikawa cells express many enzymes and structural proteins found in normal endometrium [63], along with functional steroid receptors.

Ishikawa cells treated with the SCMs of CI- and ICSI- derived embryos showed higher levels of ɑ_v_ transcript, compared to the cells treated with media alone. This suggests that human embryo secretome has factors that are capable of upregulating the expression of integrin ɑ_v_ transcript in endometrial epithelial cells. These results are in contrast to the previous observations. Our previous in-vitro studies [34] demonstrated comparable expression of ɑ_v_ integrin protein in Ishikawa cells treated with the SCM of human IVF embryos and cells treated with media alone. β_3_ protein expression, on the other hand, was found significantly higher in the cells treated with the SCM of IVF embryos, compared to these treated with media alone. The present study however, demonstrated significantly higher expression of ɑ_v_ transcript in all study groups, as compared to control group and higher β_3_ transcript levels only in ICSI group, compared to the control group. This discordance can be attributed to a different study design adopted in the present study. Firstly, in contrast to our previous study, expression of ɑ_v_β_3_ protein (a functional heterodimer) was investigated in cells treated with the secretome of single embryos, rather than a pooled secretome of different embryos of unknown quality. It is likely that the pooled conditional media of human embryos failed to cause a significant increase in the expression of ɑ_v_ protein because of potential dilution of factors causing ɑ_v_ integrin expression. Further β_3_ transcript levels were significantly increased in response to the secretome of ICSI embryos. No significant change observed in the levels of β_3_ transcript in response to the secretome of CI embryos in the present study may be attributed to discordance between RNA and protein expression for β_3_ integrin in cells treated with CI embryo secretomes. Further, it is likely that significantly higher localization of ɑ_v_β_3_ heterodimer protein observed in Ishikawa cells, treated with the secretomes of CI and ICSI derived embryos resulted from either higher expression of ɑ_v_ protein or due to preferential dimerization of ɑ_v_ and β_3_ proteins.

Interestingly, when compared within CI or ICSI groups, ɑ_v_β_3_ heterodimer localization was always higher (p < 0.05) in grade I embryo secretome treated cells than in grade II secretome treated cells. Further, a significant positive correlation between ɑ_v_β_3_ expression induced by grade I CI- and ICSI-derived embryos from sibling oocytes revealed that both, CI- and ICSI-derived embryos from sibling oocytes have the potential to induce Integrin ɑ_v_β_3_ expression. However, integrated optical density values of immunoreactive Integrin ɑ_v_β_3_ was higher in Ishikawa cells treated with the SCM from GI-CI than in cells treated with the SCM from GI-ICSI.

Mucin 1 (MUC1) is a membrane-associated protein, highly expressed in luminal and glandular epithelium on LH + 7 day [64, 65] and disappears at the site of embryo attachment in response to blastocyst-derived factors [66, 67]. Patients with the history of recurrent implantation failure are reported to have higher endometrial MUC-1 expression [68, 69]. In this study, GII-ICSI embryo group demonstrated significantly higher expression of MUC-1, compared to the control group. This increase in MUC-1 protein was paralleled by a similar increase at the transcript level. These observations hint at differential ability of CI and ICSI embryos to modulate endometrial epithelium. It has been shown that CI and ICSI can differentially influence embryonic gene expression [40, 70] More studies are warranted to elucidate the mechanism by which embryo secreted factors modify the endometrial expression of ɑ_v_β_3_ and MUC-1. Certain cytokines such as interferon gamma (IFNγ) and tumor necrosis factor alpha (TNF-α) have been shown to stimulate the expression of MUC-1 in breast epithelial cells [71], whereas transforming growth factor beta-1 (TGF-β1)-mediated upregulation of ɑ_v_β_3_ was reported in peritoneal fibroblasts [72]. The presence of these cytokines has been reported in embryo conditioned medium [73] and are known to play critical role in embryo implantation and pregnancy. Further studies are warranted to decipher whether the embryo-derived IFNγ, TNF-α and TGF-β1 modulate the expression of uterine ɑ_v_β_3_ and MUC1.

A successful implantation needs a competent embryo, a receptive endometrium and an effective cross-talk between them. It is well established that endocrine factors, endometrial pathology [74], and immunological factors [75], influence the endometrial receptivity and affect the implantation potential. However the effects of fertilization mechanisms on the implantation remain poorly understood. From the clinical ART perspectives, the observations made in this study provide new insights into possible influence of laboratory manipulation of gametes on embryo-endometrial interaction. Observations from the present study also hint at clinical opportunities to assess the embryo competence for implantation.

The strengths of this study are the use of secretomes of CI and ICSI derived embryos from sibling oocytes. To our knowledge, this approach has not been adopted to elucidate the effect of CI and ICSI on embryo quality. Since the secretomes from day 3 embryos were utilized, there is a scope to develop non-invasive biomarker to predict the embryo implantation potential. The limitations of our study are i) lack of data on the pregnancy outcome due to transfer of more than one embryo to patients either from CI or ICSI cohort ii) lack of data on embryonic gene expression due to ethical restrictions. Genetic testing of the embryos would have provided evidence on the association between the secretome and ploidy status of the embryos which was not done in our study.

## Conclusion

A cell-based *in vitro* approach, revealed that CI- and ICSI- derived embryo secretomes modulate the expression of endometrial cells *in vitro*. Therefore, it is possible that embryo-endometrial interaction and implantation post-embryo transfer are also different for CI- and ICSI- derived embryos. Since the existing data on the clinical outcome between the two insemination techniques in non-male factor infertility are contradictory, the observation made, especially in the context of ɑ_v_β_3_ protein localization, favours CI. Hence, ICSI technique should be utilized for the indication it was developed for, and not as a first-line insemination method for patients with non-male factor infertility.

## Data Availability

The data and material that support the findings of this study are available from the corresponding author upon request.
